# Highly Sensitive and Flexible Capacitive Pressure Sensors Based on Vertical Graphene and Micro-Pyramidal Dielectric Layer

**DOI:** 10.3390/nano13040701

**Published:** 2023-02-11

**Authors:** Ke Zhao, Jiemin Han, Yifei Ma, Zhaomin Tong, Jonghwan Suhr, Mei Wang, Liantuan Xiao, Suotang Jia, Xuyuan Chen

**Affiliations:** 1 State Key Laboratory of Quantum Optics and Quantum Optics Devices, Institute of Laser Spectroscopy, Collaborative Innovation Center of Extreme Optics, Shanxi University, Taiyuan 030006, China; 2 Department of Polymer Science and Engineering, School of Mechanical Engineering, Sungkyunkwan University, Suwon 16419, Republic of Korea; 3 Faculty of Technology, Natural Sciences and Maritime Sciences, Department of Microsystems, University of Southeast Norway, 3184 Borre, Norway

**Keywords:** capacitive pressure sensor, vertical graphene, micro-pyramid, high sensitivity

## Abstract

Many practical applications require flexible high-sensitivity pressure sensors. However, such sensors are difficult to achieve using conventional materials. Engineering the morphology of the electrodes and the topography of the dielectrics has been demonstrated to be effective in boosting the sensing performance of capacitive pressure sensors. In this study, a flexible capacitive pressure sensor with high sensitivity was fabricated by using three-dimensional vertical graphene (VG) as the electrode and micro-pyramidal polydimethylsiloxane (PDMS) as the dielectric layer. The engineering of the VG morphology, size, and interval of the micro-pyramids in the PDMS dielectric layer significantly boosted the sensor sensitivity. As a result, the sensors demonstrated an exceptional sensitivity of up to 6.04 kPa^−1^ in the pressure range of 0–1 kPa, and 0.69 kPa^−1^ under 1–10 kPa. Finite element analysis revealed that the micro-pyramid structure in the dielectric layer generated a significant deformation effect under pressure, thereby ameliorating the sensing properties. Finally, the sensor was used to monitor finger joint movement, knee motion, facial expression, and pressure distribution. The results indicate that the sensor exhibits great potential in various applications, including human motion detection and human-machine interaction.

## 1. Introduction

With the development of health-monitoring applications, flexible sensors are being required in growing number [1]. The existing flexible tactile sensors can be divided according to their working mechanism, including piezoresistive [2,3], capacitive [4,5,6,7,8], triboelectric [9,10], and piezoelectric [11,12,13,14,15] sensors. Among these sensors, capacitive sensors with a dielectric layer sandwiched between two electrodes exhibit the advantages of a simple structure, facile manufacturing, low energy consumption, and static/dynamic detection. Therefore, flexible capacitive pressure sensors have attracted extensive attention in tactile sensing [16,17], environmental detection [18,19], and human motion monitoring [20,21,22]. High sensitivity is key to achieving high-pressure resolution, which is essential for simplifying signal processing [23]. Additionally, the sensitivity of capacitive pressure sensors should be continuously improved to meet the requirements of increasingly complex applications.

The active electrode material is essential for determining the properties of flexible capacitive pressure sensors. Many conductive materials, such as carbon nanotubes [24,25,26], graphene [27,28], and metal nanoparticles [29,30,31], have been used in flexible pressure sensors. Among these, graphene and its derivatives demonstrate great potential, owing to their excellent mechanical properties and adjustable microstructures [32,33,34,35]. To meet the requirements for wearable electronics and electronic skins, it is necessary to develop a flexible graphene-based pressure sensor with high sensitivity. Vertical graphene (VG), also known as graphene nanowalls, is a graphene derivative with vertically oriented graphene flakes grown on a planar carbon film. Recently, Yang et al. developed VG as an active electrode material in a capacitive pressure sensor. By hybridizing the conformally micro-structured electrodes with dielectrics, enhanced sensitivity, pressure range, and stability were achieved [36]. However, the advantages of VG over traditional two-dimensional graphene and the effect of the VG morphology on the sensing performance have not yet been studied.

In addition to the active electrode material, the dielectric layer is crucial for sensing performance. The sensitivity of conventional capacitive pressure sensors with a planar dielectric layer cannot be further improved because of the limited deformation capability of the dielectric layer. Recent reports have demonstrated that the sensing performance of capacitive pressure sensors can be greatly improved by the microstructural engineering of the dielectric layers [37,38] with topographies, such as micro-domes [39], micro-pyramid arrays [40,41,42], micro-capillaries [43], and layered structures [44,45]. In these cases, under a low-pressure load, only the tips of the microstructures contact the electrode surface, thereby amplifying the deformation of the dielectrics and enhancing the sensing performance.

In this study, flexible capacitive pressure sensors with high sensitivity were fabricated with VG electrodes and a pyramidal polydimethylsiloxane (PDMS) dielectric layer (VG-P sensor). VG, as a conductive material for flexible capacitive electrodes, was synthesised via a plasma-enhanced chemical vapor deposition (PECVD) process. A dielectric PDMS film with pyramid arrays was prepared using a template-casting process. By controlling the VG morphology and size of the PDMS pyramids, the sensitivity of the sensor reached as high as 6.04 kPa^−1^ under 0.1–1 kPa, and 0.69 kPa^−1^ under 1–10 kPa. Finite-element simulations indicated that the micro-pyramids concentrated the pressure and improved the deformability of the dielectric layer. Therefore, the sensor exhibited excellent sensitivity, suggesting promising prospects in the fields of health detection, expression recognition, and human-machine interface.

## 2. Materials and Methods

### 2.1. Preparation of Sensors

VG was synthesised by using a method similar to that in our previous studies [46]. First, quartz sheets (2 × 2 cm^2^) were cleaned with deionised (DI) water, alcohol, and acetone under ultrasonication. They were then placed in a tubular furnace for VG growth via plasma-enhanced chemical vapor deposition (PECVD). Two standard cubic centimeter per minute (sccm) of H_2_ and 6 sccm of C_2_H_2_ were introduced into the furnace under the temperature and power conditions shown in Table S1 for 1 h at a pressure of 1 mTorr. After naturally cooling to room temperature, the VG films with different densities were grown on the quartz surface. A silicone elastomer (Dow Corning Sylgard 184, Auburn, AL, USA) was mixed in a ratio of 10:1 to prepare a PDMS solution. The PDMS solution was poured onto the VG surface, forming a 400 µm thick film, and then peeled off from the quartz surface after curing at 80 °C for 2 h. Thus, VG/PDMS flexible electrodes were fabricated.

Second, a Si template with inverted micro-pyramid arrays was used as the substrate for the PDMS dielectric layer with micro-pyramid structures. The pyramid pattern was prepared via a wet etching process on four-inch Si wafers, which were then chemically and anisotropically etched with potassium hydroxide. The micropatterned Si template was cleaned using alcohol and acetone under ultrasonication. After spin-coating and drying on the Si template, the PDMS dielectric layer with micro-pyramid arrays was peeled off. Micro-pyramids with different side lengths and intervals, i.e., 3, 5, 10, and 20 µm, were prepared. The thickness of the dielectric film was approximately 300 µm. Finally, flexible capacitive pressure sensors were successfully fabricated by combining the upper and lower layers of the VG/PDMS film with the dielectric layer in a sandwich-like structure.

### 2.2. Characterization and Measurements

The morphologies of the samples were characterised via scanning electron microscopy (SEM; HITACHI SU-8010, Hitachi Limited, Japan). A Horiba Scientific LabRAM HR Evolution system with a laser operating at 532 nm was used to record the Raman spectra. The pressure was applied using an electric tensile pressure tester (ZQ-990B, ZHIQU, Dongguan, China). A Keysight E4980A Precision LCR Meter was used to record the capacitance values. The four-point probe method (Keithley 2420I−V, Tektronix, Shanghai, China) was used to measure the sheet resistance of the VG.

The pressure was calculated as *P = F/A*, where *F* is the force applied by the electric tensile pressure tester and *A* is the area of the sensor. The sensor size was 2 cm × 0.5 cm (length × width), and its area was 1 cm^2^. The sensitivity is defined as *S* = (Δ*C*/*C*_0_)/Δ*P*, where Δ*C* is the capacitance change under pressure, *C*_0_ is the initial capacitance, and Δ*P* is the pressure change.

COMSOL Multiphysics 5.6 software was used for finite element analysis (FEA). A pyramid with a width of 20 μm and height of 14.14 μm was used in the analysis. In the pyramid arrays, the interval between the pyramids was 20 μm. In the simulation, a pressure loading of 10 kPa was applied to the top surface of the model. The pyramid tips were pressed against the electrode layer for deformation. The model was analyzed using a three-dimensional deformation strain physical model.

## 3. Results and Discussion

**Figure 1:**
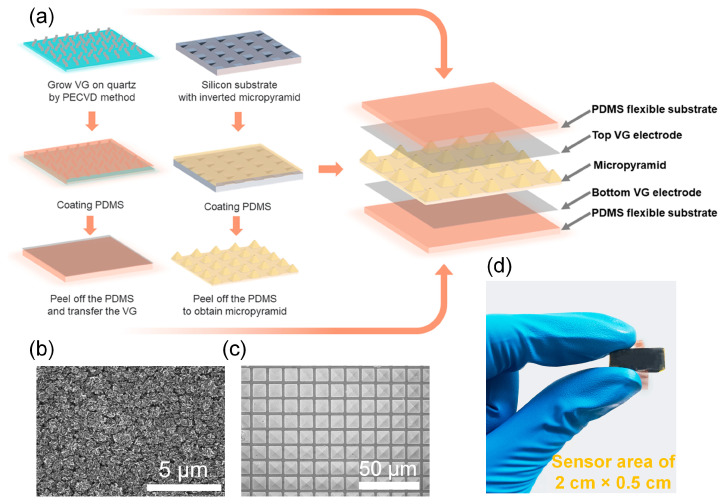
(**a**) Assembly process and configuration of the flexible capacitive pressure sensor. (**b**) Scanning electron microscopy (SEM) image of the vertical graphene (VG) electrode. (**c**) SEM image of the micro-pyramidal polydimethylsiloxane (PDMS) dielectric layer. (**d**) Photograph of a flexible capacitive pressure sensor.

The fabrication process of the flexible capacitive pressure sensor is shown in Figure 1a. The sensor comprised a micro-pyramidal PDMS dielectric layer and two VG electrodes in a sandwich-like structure. The VG was grown on a quartz plate via the PECVD method; thereafter, the quartz surface was completely covered by vertically oriented graphene sheets (Figure 1b). After spin-coating the PDMS film and peeling off the cured PDMS film, a VG/PDMS film was obtained as the electrode of the capacitive sensor. The flexible PDMS film served as the supporting and encapsulating layers of the VG. Because the VG film consists of a planar bottom carbon layer and vertically oriented graphene sheets [47], its rough surface is beneficial for adhesion to the PDMS film. The dielectric layer, a pyramidal PDMS film (Figure 1c), was obtained by using a film casting process on an inverted pyramid-patterned silicon wafer. A capacitive flexible pressure sensor was fabricated by connecting two copper conductors, as shown in Figure 1d.

**Figure 2:**
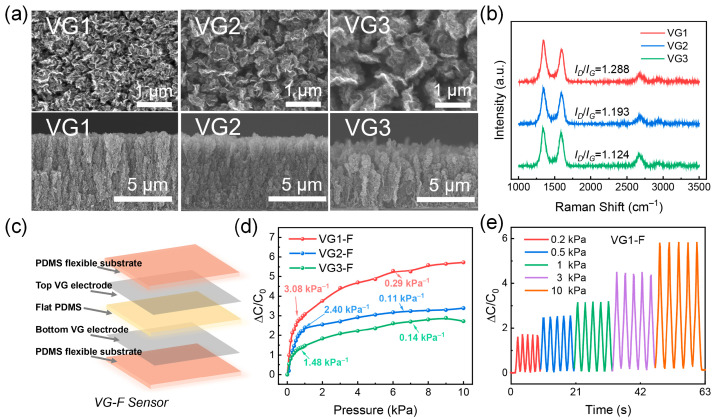
(**a**) Top-view and cross-sectional scanning electron microscopy images of the VG1, VG2, and VG3 films on quartz. (**b**) Raman spectra of the VG1, VG2, and VG3 films. (**c**) Schematic illustration of the vertical graphene (VG) based pressure sensor using a flat polydimethylsiloxane (PDMS) dielectric layer. (**d**) Capacitance response of the VG1-F, VG2-F, and VG3-F sensors at pressures of 0–10 kPa. (**e**) Capacitance response of the VG1-F sensor at pressures of 0–10 kPa for five pressure loading/release cycles.

To reveal the effect of the VG morphology on the sensor properties, three VG films with different densities were synthesised by controlling the deposition conditions, which are denoted as VG1, VG2, and VG3. The detailed growth conditions of the VG films with different densities are listed in Table S1. The SEM images in Figure 2a reveal that VG is composed of multilayer graphene with a relatively thick nanosheet. Furthermore, the top-view and cross-sectional SEM images show that VG1 is composed of stacked small nanoflakes, exhibiting the highest density. The nanoflakes in VG2 are larger than those in VG1 and exhibit a relatively loosened structure. VG3 has the largest VG nanosheets and exhibits the lowest density. The densities of the VG films were also obtained from the SEM images using ImageJ software, as shown in Figure S1. The results show that the proportion of VG1 in the height range of 4.6–6.9 μm is 11%, which is significantly higher than those of VG2 (6 %) and VG3 (3 %). Meanwhile, the proportion of VG1 in the height region of 2.3–4.6 μm is 47 %, which is also higher than those of VG2 (31%) and VG3 (28%). The high proportions in certain height ranges indicate the high density of the VG films and demonstrate that VG1 exhibits the highest density among the VG films.

Raman spectra also confirmed the successful growth of VG1, VG2, and VG3 by the representative peaks of D, G, and 2D appearing at 1350, 1608, and 2680 cm^−1^, respectively (Figure 2b). The intensity ratios of the D and G peaks (*I_D_*/*I_G_*) can be used to characterise the level of disorder and the defects in graphene. The *I_D_*/*I_G_* values of VG1, VG2, and VG3 were 1.288, 1.193, and 1.124, respectively, indicating that VG1 possesses the highest defect density. This is because VG1 is grown at a low temperature with high plasma power, which has been demonstrated to cause more defects during VG growth [47].

The low sheet resistance of the VG films (Figure S2) implies that they are suitable for electrode applications. Among them, VG1 has the lowest sheet resistance, indicating its superior electrical conductivity. VG1, VG2, and VG3 were transferred to flexible PDMS and assembled with a flat PDMS dielectric layer to obtain the VG-based pressure sensors VG1-F, VG2-F, and VG3-F, respectively. The construction of the VG-F sensor is shown in Figure 2c. The sensing response and sensitivity of the VG-based sensors in Figure 2d show that VG1-F exhibits the highest sensitivity of 2.32 kPa^−1^ under 0–1 kPa, which is higher than those of VG2-F (2.23 kPa^−1^) and VG3-F (1.04 kPa^−1^). VG1-F also exhibits the highest sensitivity of 0.29 kPa^−1^ within 1–10 kPa, which is higher than those of the other two sensors (0.11 kPa^−1^ and 0.14 kPa^−1^; Table S2). Compared with previous studies [23,43,48,49], our VG-F flexible capacitive pressure sensor possesses desirable sensitivity. Furthermore, the step response of the VG1-F sensor in the pressure range of 0–1 kPa was analyzed. The results show instant capacitance responses to pressure (Figure S3). Figure 2e shows the cyclic response curves of VG1-F at 0.2, 0.5, 1, 3, and 10 kPa for five pressure loading/release cycles. The sharp and stable response peaks of the pressure indicate the instant detection and exceptional stability of VG1-F.

**Figure 3:**
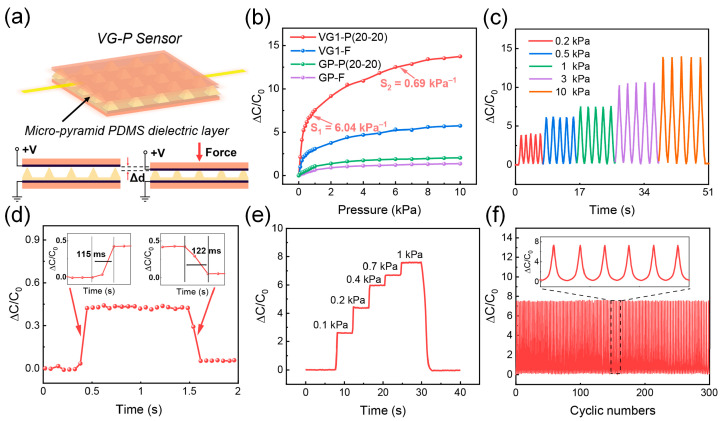
(**a**) Schematic configuration of the micro-pyramidal capacitive pressure sensor. (**b**) Capacitance response of the VG1-P(20-20), VG1-F, GP-P(20-20), and GP-F sensors. (**c**) Real-time responses of the VG1-P(20-20) sensor to pressures of 0.2, 0.5, 1, 3, and 10 kPa. (**d**) Real-time response of the VG1-P(20-20) sensor to an ultralow pressure of 20 Pa. The response time upon loading and unloading is shown in the insets. (**e**) Capacitance response under different pressure loadings at 0–1 kPa. (**f**) Cyclic test of the VG1-P(20-20) sensor for 300 cycles under an applied pressure of 1 kPa; the insets show the 150th to 155th cycles.

To further boost the sensitivity of the VG1-based capacitive pressure sensor, a PDMS dielectric layer with micro-pyramid patterns was introduced to the sensor, as shown in Figure 3a. The sensors with VG1 electrodes and a micro-pyramidal PDMS dielectric layer are denoted as VG1-P. To investigate the sensitivities of the VG1-P sensors, a VG1-P(20-20) sensor was fabricated, in which the first number indicates the pyramid size (20 μm) and the second number represents the interval (20 μm) between the pyramids (Figure S4). Figure 3b compares the capacitance responses of VG1-F and VG1-P(20-20). VG1-P(20-20) achieved an exceptional sensitivity of 6.04 kPa^−1^, which is considerably higher than that of VG1-F, demonstrating the advantages of the micro-pyramid patterned dielectric layer. Moreover, to study the effect of the micro-pyramid patterns on the sensing performance of the VG1-P sensors, we fabricated and tested various sensors with different pyramid sizes and intervals, as listed in Table S3. Figure S5 shows the capacitance-response curves and the sensitivities of the VG1-P sensors, indicating that VG1-P(20-20) achieved the highest sensitivity among the VG1-P sensors. The capacitance response of the VG1-P sensors exhibits two stages. During the first stage (0–1 kPa), an exceptional sensitivity of 6.04 kPa^−1^ is observed. Meanwhile, the second stage (1–10 kPa) provides a sensitivity of 0.69 kPa^−1^. Furthermore, the sensitivity values reveal that as the size and interval of the pyramids increase, the sensitivity of the VG1-P sensors improves.

Furthermore, the advantages of VG over traditional 2D carbon films were investigated by comparing VG1-P and VG1-F with capacitive sensors based on graphite paper electrodes, as shown in Figure 3b. The sensitivity values are listed in Table S4. The results show that the VG-based sensors exhibit a significantly stronger capacitance response to pressure. Additionally, a graphite paper-based sensor with a micro-pyramidal PDMS dielectric layer (GP-P(20-20)) exhibits better sensing performance than that with a flat dielectric layer (GP-F). These results demonstrate the superiority of the VG electrodes and micro-pyramidal dielectrics. In addition, when compared with the results in the literature, the pyramid structure of the dielectric layer is superior to other geometries, as shown in Table S5 [43,50,51,52,53,54,55,56,57].

Furthermore, we tested the real-time response of the VG1-P(20-20) sensor under loading/unloading pressures of 0.2, 0.5, 1, 3, and 10 kPa for five cycles at each pressure (Figure 3c). The results show that the flexible sensor exhibits stable responses and that the relative capacitance change values are in accordance with the results shown in Figure 3b. To further investigate the response speed of the VG1-P(20-20) sensor, a pressure of 0.02 kPa was loaded (Figure 3d). The capacitance response increased rapidly within 126 ms and remained stable under pressure loading. After withdrawing the pressure, the capacitance response promptly recovered to the initial value within 189 ms. The instant response suggests the promising potential of the VG1-P(20-20) sensor for dynamic consecutive pressure detection. Figure 3e shows VG1-P(20-20) under different pressure loadings. The VG1-P(20-20) sensor was pressed step by step from 0.1 to 1 kPa and then released. As a result, the signal increased in a step-wise manner and then returned to the initial position, indicating that the sensor was responsive to different pressures and exhibited desirable holding stability.

To investigate the mechanical durability of the sensor, VG1-P(20-20) was subjected to 300 compression/release cycles at a pressure of 1 kPa. As shown in Figure 3f, the signals are stable, and no fatigue can be observed. The inset in Figure 3f shows that the single peaks maintained the same shape and intensity, confirming the high repeatability and durability of the sensor. Comparing the VG1-P(20-20) sensor with the reported sensors, as listed in Table S6 [17,21,56,58,59,60,61,62,63,64,65,66], the former exhibits superior sensing performance.

**Figure 4:**
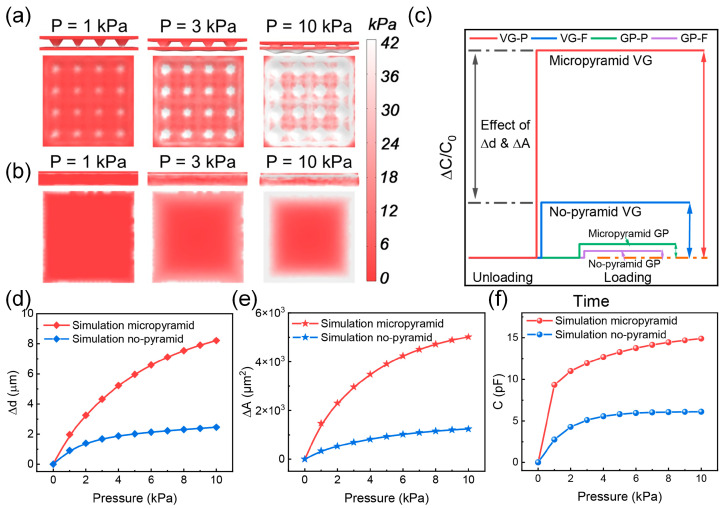
(**a**) Finite-element simulation of the pressure distribution and deformation of the micro-pyramidal polydimethylsiloxane (PDMS) dielectric layer. (**b**) Finite-element analysis of the pressure distribution and deformation of the flat PDMS dielectric layer. (**c**) Relative capacitance variations of the VG1-P(20-20), VG1-F, GP-P(20-20), and GP-F pressure sensors under a pressure of 10 kPa. (**d**) Simulated electrode distance change versus pressure. (**e**) Simulated effective contact area change versus pressure. (**f**) Simulated capacitance change versus pressure.

To elucidate the sensing mechanism of the sensor, FEA was conducted to simulate the pressure distribution of the micro-pyramidal and flat PDMS dielectric layers under different external pressures, as shown in Figure 4a,b. Pyramid heights of 14.14 μm were used in the simulation and they were calculated based on the angle of the (100) and (111) planes of the Si crystal (54.74°). Figure 4a shows that only the tips of the pyramids contact the bottom electrode in the initial state. The pressure is concentrated on the pyramid tips, resulting in their distortion. As the distortion increases, the contact area between the pyramids and the electrode increases, and the distance between the electrodes decreases correspondingly. However, in the case of the VG-F sensor, the pressure is distributed uniformly on the flat dielectric layer (Figure 4b), providing a much smaller change in distance than that provided by the VG-P sensor.

Figure 4c depicts the simulation results of the capacitance response of the VG1-P(20-20), VG1-F, GP-P(20-20), and GP-F pressure sensors under pressure loading. The sensor with the VG electrode and micro-pyramid dielectric layer exhibits a superior capacitance response to those with the graphite paper electrode or flat dielectric layer, indicating a higher sensitivity under an identical applied pressure. According to the formula for the capacitance, *C = ε_0_ε_r_A/d*, where *ε_0_* is the vacuum permittivity, *ε_r_* is the initial relative permittivity, A is the electrode area, and d is the distance between the two electrodes. The sensing mechanism of the capacitive pressure sensor relies on the distance between the electrodes and the contact area. Figure 4d,e show the dependence of the distance and contact area change on the applied pressure of 0–10 kPa for sensors with flat and micro-pyramidal dielectric layers. The micro-pyramidal dielectric layer shows a higher area change and lower distance change, which led to a greater capacitance change. Figure 4f shows the simulated capacitance data plotted with curves composed of two linear regions, similar to the experimental results in Figure 3b. Therefore, the micro-pyramidal configuration of the dielectric layer is highly effective in enhancing the sensing performance of the sensors.

**Figure 5:**
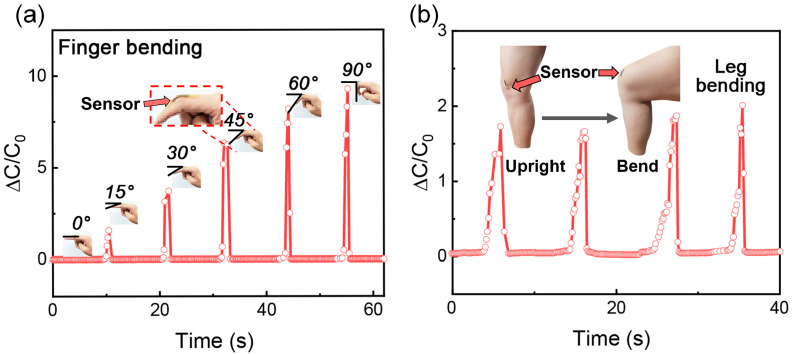
(**a**) Sensing performance of the VG1-P(20-20) sensor for finger bending. (**b**) Sensing performance of the VG1-P(20-20) sensor in leg bending.

Owing to the excellent skin adhesion of the flexible capacitive pressure sensor, the VG1-P(20-20) sensor is compatible with the human body for posture recognition. The sensor was attached to the knuckle of the index finger of the test subject, allowing it to respond to various bending conditions (Figure 5a). Evidently, the sensor accurately responded to the angle changes in the bending of the index finger, and the output signal increased with an increase in the bending angle. Similarly, a sensor was attached to the knee, and the output capacitance signal was used to distinguish whether or not the leg was bent, as shown in Figure 5b. The output reliability of the sensor was verified by four consecutive bends with an accurate and stable response, indicating the excellent mechanical and sensing properties of the sensor. Therefore, the VG1-P(20-20) sensor shows great potential for human movement and rehabilitation, motion monitoring, and human-computer interface.

**Figure 6:**
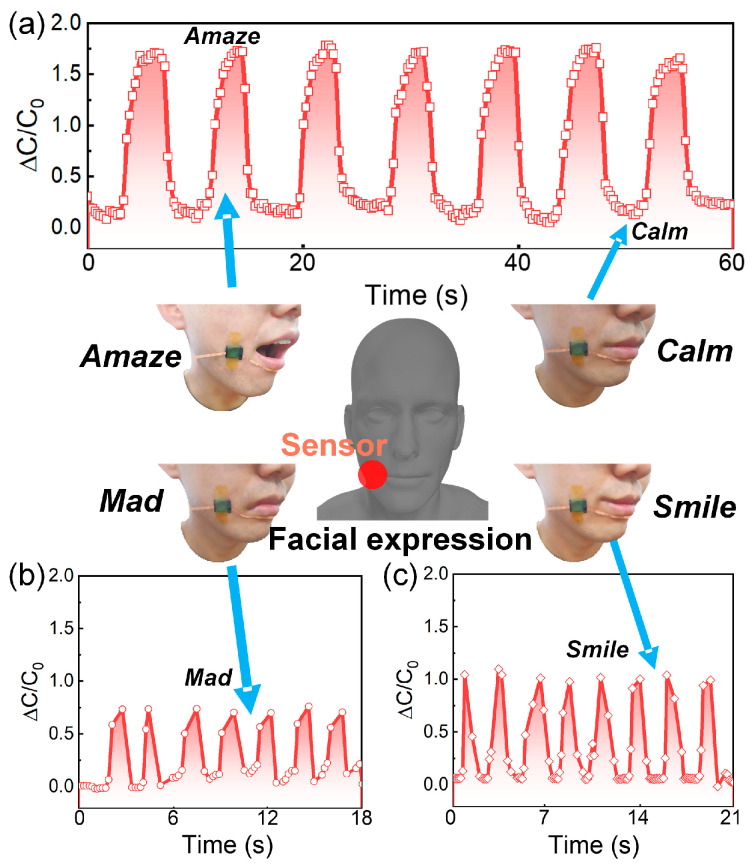
Application of the VG1-P(20-20) sensor to identify facial expressions: (**a**) amazed and calm, (**b**) angry, and (**c**) smiling.

Furthermore, the VG1-P(20-20) sensor was applied to detect subtle human facial expressions. As shown in Figure 6, the sensor was placed on the right cheek of the testee, and its response was triggered by the movement of the mimetic muscles. As a result, the flexible capacitive pressure sensor responded stably to the facial expressions of amazed, calm, angry, and smiling, owing to its high sensitivity, excellent stability, good flexibility, low detection limit, and fast response. The results indicate that the sensor exhibits considerable potential for the real-time monitoring and quantitative evaluation of complex facial expressions.

**Figure 7:**
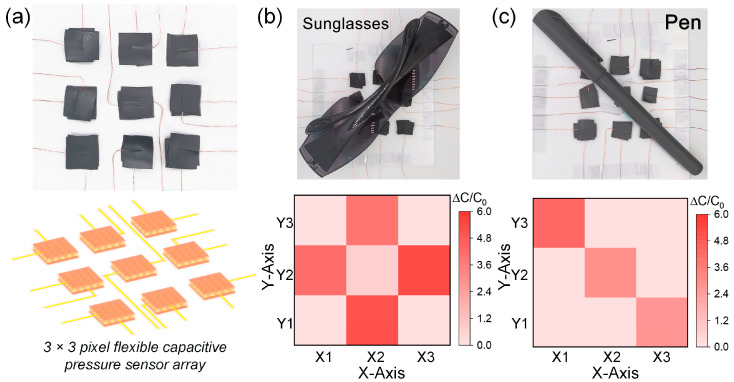
(**a**) Schematic photo and illustration of a 3 × 3 pixel flexible capacitive pressure sensor array. (**b**) Photo of a pair of sunglasses on the sensor array and the corresponding signal intensity distribution. (**c**) Photo of a pen on the sensor array and the corresponding signal intensity distribution.

The capability to recognize pressure distribution is key to artificial e-skin applications in the fields of intelligent robots and prosthetics. Based on this, a 3 × 3 pixel flexible capacitive pressure sensor array was designed and assembled to detect the pressure distribution. As shown in Figure 7a, the size of each capacitive sensor was 1.5 × 1.5 cm^2^, and the size of the sensing array was 8 × 8 cm^2^. Copper wires were connected to the upper and lower electrodes, acting as conductors. Figure 7b,c shows the pressure distribution of the sunglasses and pen placed on the sensing array, respectively. The pressure distribution was reflected by the capacitance changes in each pixel. Pressure mapping of the relative capacitance changes shows that the sensor array can effectively distinguish the placement position and pressure of objects with different shapes, thereby demonstrating great potential in soft robotics and electronic skin applications.

## 4. Conclusions

In summary, a flexible capacitive pressure sensor with high sensitivity was designed and fabricated based on VG electrodes and a micro-pyramidal dielectric layer. Compared with the traditional planar electrode, the sensor sensitivity was improved using three-dimensional VG. Meanwhile, the micro-pyramid design in the dielectric layer enhanced the deformation effect of the dielectric layer under pressure and further improved the sensitivity of the sensor. The sensor achieved high sensitivities of 6.04 kPa^−1^ under 0–1 kPa and 0.69 kPa^−1^ under 1–10 kPa. The flexible capacitive pressure sensor prepared in this study exhibits the advantages of facile manufacturing, high sensitivity, and reliable stability, and showing great promise for a prominent range of applications, including expression recognition, human-computer interaction, and health monitoring.

## Data Availability

Not applicable.

## Supplementary Materials

The following supporting information can be downloaded at: https://www.mdpi.com/article/10.3390/nano13040701/s1, Figure S1: Height distribution ratios of the VG1, VG2, and VG3 films analyzed using Image J software; Figure S2: Sheet resistance of the VG1, VG2, and VG3 films; Figure S3: Step response of the VG1-F sensor at the pressure of 0–1 kPa; Figure S4: Illustration of the size and interval of the micro-pyramids in the PDMS dielectric layer; Figure S5: Relative capacitance changes of the VG1-based capacitive pressure sensors with different micro-pyramid sizes and intervals at a pressure range of 0–10 kPa; Table S1: Growth conditions of the VG films with different morphologies; Table S2: Comparison of the sensitivities of the VG1-F, VG2-F, and VG3-F sensors; Table S3: Comparison of the sensitivities of the VG-based sensors with different micro-pyramid sizes and intervals; Table S4: Comparison of the sensitivities of the VG-based sensors and graphite paper-based sensors with/without micro-pyramidal PDMS dielectric layer; Table S5: Comparison of the performances of the pyramid structured interfaces with other reported geometries. References [43,50,51,52,53,54,55,56,57] are cited in the supplementary materials. Table S6: Comparison of the performances of the VG based sensor with other pressure sensors in the literature. References [17,21,56,58,59,60,61,62,63,64,65,66] are cited in the supplementary materials.
