# Supplementary material for: Highly Sensitive and Flexible Capacitive Pressure Sensors Based on Vertical Graphene and Micro-Pyramidal Dielectric Layer
Source: Nanomaterials (Basel). 2023 Feb 11;13(4):701. doi: 10.3390/nano13040701 (PMC9962134; doi:10.3390/nano13040701)
Supplement: Supplementary file 1 [file nanomaterials-13-00701-s001.zip › nanomaterials-2174675-supplementary.pdf]

nanomaterials

Supplementary Materials

Highly sensitive and flexible capacitive pressure sensors based on vertical graphene and micro-pyramidal dielectric layer

Ke Zhao[1,†], Jiemin Han[1,†], Yifei Ma[1,*], Zhaomin Tong[1], Jonghwan Suhr[2], Mei Wang[1,*], Liantuan Xiao[1], Suotang Jia[1], and Xuyuan Chen[1,3]

1 State Key Laboratory of Quantum Optics and Quantum Optics Devices, Institute of Laser Spectroscopy, Collaborative Innovation Center of Extreme Optics, Shanxi University, Taiyuan, 030006, China
2 Department of Polymer Science and Engineering, School of Mechanical Engineering, Sungkyunkwan University, 16419 Suwon, Republic of Korea
3 Faculty of Technology, Natural Sciences and Maritime Sciences, Department of Microsystems, University of Southeast Norway, Borre 3184, Norway
* Correspondence: author: mayifei@sxu.edu.cn (Y.M.); wangmei@sxu.edu.cn (M.W.)
† These authors contribute equally to this work.

Table S1. Growth conditions of the VG films with different morphologies.

	Growth Time (min)	Temperature (℃)	Plasma Power (W)
VG1	60	700	280
VG2	60	800	180
VG3	60	800	280

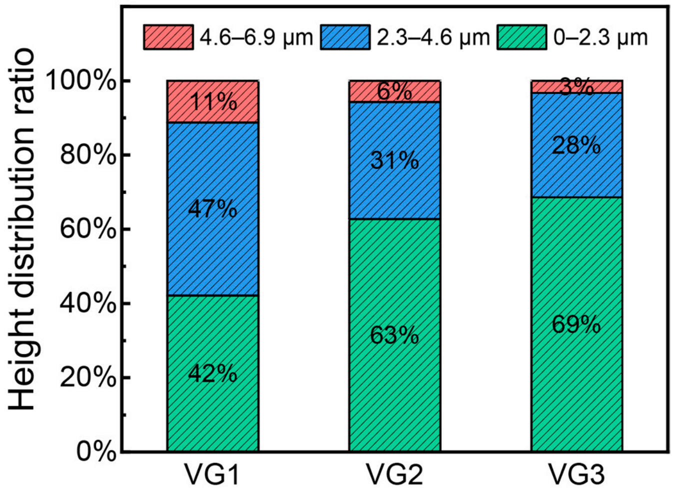

Figure S1. Height distribution ratios of the VG1, VG2, and VG3 films analyzed using Image J software.

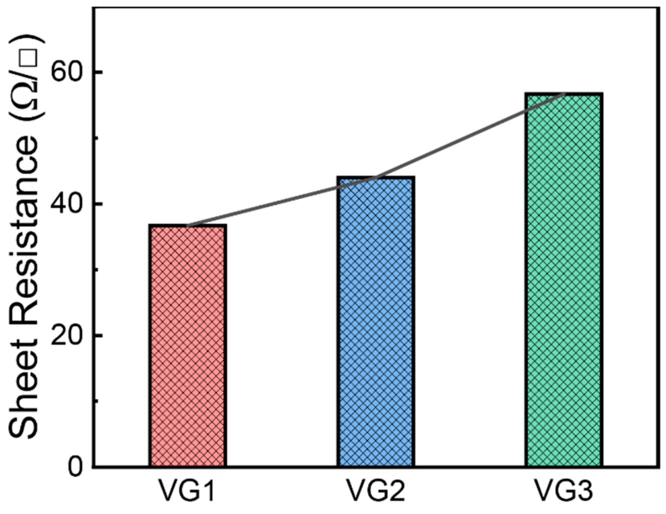

Figure S2. Sheet resistance of the VG1, VG2, and VG3 films.

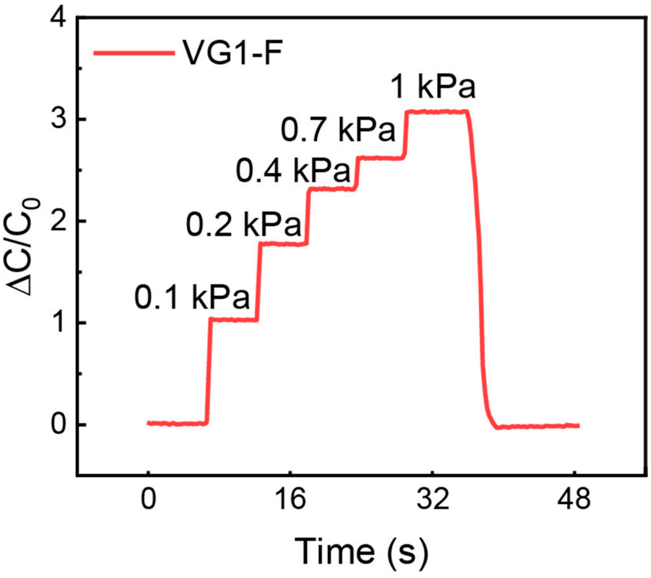

Figure S3. Step response of the VG1-F sensor at the pressure of 0–1 kPa.

Table S2. Comparison of the sensitivities of the VG1-F, VG2-F, and VG3-F sensors.

	Sensitivity of 0–1 kPa (kPa^{-1})	Sensitivity of 1–10 kPa (kPa^{-1})
**VG1-F**	2.32	0.29
VG2-F	2.23	0.11
VG3-F	1.04	0.14

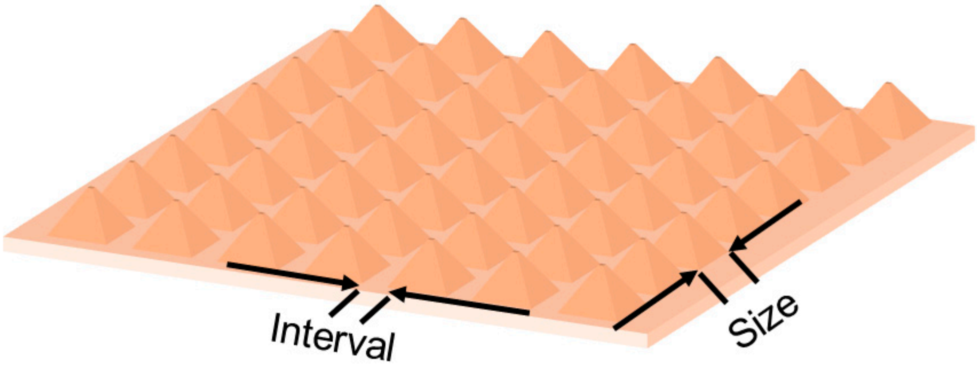

Figure S4. Illustration of the size and interval of the micro-pyramids in the PDMS dielectric layer.

Table S3. Comparison of the sensitivities of the VG-based sensors with different micro-pyramid sizes and intervals.

	Sensitivity in 0–1 kPa (kPa^{-1})	Sensitivity in 1–10 kPa (kPa^{-1})
VG1-F	2.32	0.29
VG1-P(3-3)	2.31	0.33
VG1-P(3-5)	2.40	0.35
VG1-P(5-3)	2.24	0.37
VG1-P(5-5)	2.53	0.37
VG1-P(3-10)	3.21	0.37
VG1-P(10-3)	3.55	0.41
VG1-P(10-10)	4.79	0.54
VG1-P(20-10)	5.76	0.59
**VG1-P(20-20)**	6.04	0.69

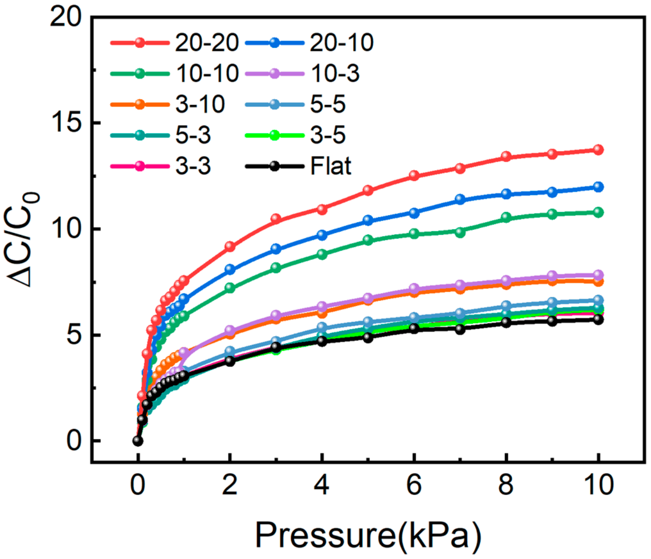

Figure S5. Relative capacitance changes of the VG1-based capacitive pressure sensors with different micro-pyramid sizes and intervals at a pressure range of 0–10 kPa.

Table S4. Comparison of the sensitivities of the VG-based sensors and graphite paper-based sensors with/without micro-pyramidal PDMS dielectric layer.

	**Sensitivity in 0–1 kPa (kPa^{-1})**	**Sensitivity in 1–10 kPa (kPa^{-1})**
GP-F	0.59	0.08
GP-P(20-20)	0.91	0.11
VG1-F	2.32	0.29
**VG1-P(20-20)**	**6.04**	**0.69**

Table S5. Comparison of the performances of the pyramid structured interfaces with other reported geometries.

Geometric shapes	Sensitivity (kPa^{-1})	Detection range	ref
Micropillar	5.3<br>0.4	<1 kPa<br>1-10 kPa	[50]
Porous Polydimethylsiloxane Elastomer	0.717<br>0.360<br>0.200	0-0.05 kPa<br>0.05-1 kPa<br>1-3 kPa	[51]
Interlocked microdome	0.715	0-5 kPa	[52]
Micro-nano hierarchical structures	0.055	0.5-10 kPa	[53]
M-Tooth Hybrid Micro-Structure-Based	0.5136<br>0.0847<br>0.0247	0-1 kPa<br>1-10 kPa<br>10-40 kPa	[54]
Microcylindrical structures	5<br>1.17<br>0.04	0-5 kPa<br>5-7.5 kPa<br>7.5-40 kPa	[55]
Microtower	1.194<br>0.077	<2 kPa<br>2-15 kPa	[56]
Tilted micropillar	0.42<br>0.04	<1.5 kPa<br>5-20 kPa	[43]
Wavy	0.182<br>0.077<br>0.015	0-5 kPa<br>5-10 kPa<br>10-40 kPa	[57]
**Our pyramid work**	**6.04**<br>**0.69**	**0-1 kPa**<br>**1-10 kPa**	**This work**

Table S6. Comparison of the performances of the VG based sensor with other pressure sensors in the literature.

	Sensitivity (kPa^{-1})	Detection range	ref
Au/PDMS/PS	0.815	0-1.5 kPa	[17]
AgNWs/PDMS	1.194<br>0.077	0-2 kPa<br>2-14 kPa	[56]
Graphene	1.25	0-25 kPa	[58]
PDMS/Ag Nanowire	0.0178<br>0.0057	0-16 kPa<br>16-100 kPa	[59]
Graphite/PDMS	0.62<br>0.28<br>0.06	0-2 kPa<br>2-6 kPa<br>6-10 kPa	[60]
CNTs/PDMS	2.9	0-0.85 kPa	[21]
Carbon fibers (Honeycombfabrics)	0.045	0-10 kPa	[61]
PS/graphene/MWCNTs	0.062	0-4.5 kPa	[62]
rGO/PDMS	0.002	0-10 kPa	[63]
CNT/Ecoflex	0.601	0-5 kPa	[64]
AgNWs/PDMS	2.04	0-2 kPa	[65]
Al/Porous PDMS	4.99	0-1 kPa	[66]
**VG1-P(20-20)**	**6.04**<br>**0.69**	**0-1 kPa**<br>**1-10 kPa**	**This work**
